# Human cytomegalovirus reactivation from latency: validation of a “switch” model in vitro

**DOI:** 10.1186/s12985-016-0634-z

**Published:** 2016-10-22

**Authors:** Maria-Cristina Arcangeletti, Rosita Vasile Simone, Isabella Rodighiero, Flora De Conto, Maria-Cristina Medici, Clara Maccari, Carlo Chezzi, Adriana Calderaro

**Affiliations:** Department of Clinical and Experimental Medicine, Unit of Microbiology and Virology, University of Parma, Viale A. Gramsci, 14, Parma, 43126 Italy

**Keywords:** HCMV, Latency, Reactivation, THP-1 monocytes, THP-1 differentiation

## Abstract

**Background:**

Human cytomegalovirus (HCMV) is an opportunistic pathogen leading to severe and even fatal diseases in ‘at-risk’ categories of individuals upon primary infection or the symptomatic reactivation of the endogenous virus. The mechanisms which make the virus able to reactivate from latency are still matter of intense study. However, the very low number of peripheral blood monocytes (an important latent virus reservoir) harbouring HCMV DNA makes it very difficult to obtain adequate viral quantities to use in such studies.

Thus, the aim of the present study was to demonstrate the usefulness of human THP-1 monocytes, mostly employed as HCMV latent or lytic infection system, as a reactivation model.

**Methods:**

THP-1 monocytes were infected with HCMV TB40E strain (latency model) at multiplicities of infection (MOI) of 0.5, 0.25 or 0.125. After infection, THP-1 aliquots were differentiated into macrophages (reactivation model). Infections were carried out for 30 h, 4, 6 and 7 days. Viral DNA evaluation was performed with viable and UV-inactivated virus by q-Real-Time PCR. RNA extracted from latency and reactivation models at 7 days post-infection (p.i.) was subjected to RT-PCR to analyse viral latency and lytic transcripts. To perform viral progeny analysis and titration, the culture medium from infected THP-1 latency and reactivation models (7 days p.i.) was used to infect human fibroblasts; it was also checked for the presence of exosomes.

For viral progeny analysis experiments, the Towne strain was also used.

**Results:**

Our results showed that, while comparable TB40E DNA amounts were present in both latent and reactivation models at 30 h p.i., gradually increased quantities of viral DNA were only evident in the latter model at 4, 6, 7 days p.i.. The completion of the lytic cycle upon reactivation was also proved by the presence of HCMV lytic transcripts and an infectious viral yield at 7 days p.i.

**Conclusions:**

Our data demonstrate the effectiveness of THP-1 cells as a “switch” model for studying the mechanisms that regulate HCMV reactivation from latency. This system is able to provide adequate quantities of cells harbouring latent/reactivated virus, thereby overcoming the intrinsic difficulties connected to the *ex vivo* system.

**Electronic supplementary material:**

The online version of this article (doi:10.1186/s12985-016-0634-z) contains supplementary material, which is available to authorized users.

## Background

Human cytomegalovirus (HCMV), the prototype member of the *β*-herpesviruses [1], is a ubiquitous and widespread agent that infects the majority of the population during early childhood [2]. It establishes a lifelong latency from which it can reactivate [3–7]. Although HCMV primary infection and reactivation are asymptomatic in immunocompetent individuals, they can cause serious diseases with multi-organ involvement and even death in ‘at-risk’ categories of individuals, such as congenitally infected newborns and subjects with a deficient immune system mostly due to iatrogenic (e.g. organ transplant patients) or acquired (e.g. HIV-infected subjects) causes [8–18].

Concerning HCMV latent reservoirs, peripheral blood monocytes have been shown to be one of the most relevant cell populations harbouring latent HCMV in vivo; furthermore, HCMV latency sites have also been identified within hematopoietic stem cells in the bone marrow, particularly in undifferentiated cells of the myeloid lineage [19–27].

Unfortunately, *ex vivo* studies on HCMV latency have been severely hampered by the low frequency of viral genome-positive mononuclear cells (around only 0.01 %) and their low HCMV DNA content [28–32].

Several studies provide evidence indicating that the differentiation of monocytes into macrophages in vivo could represent a key event triggering the reactivation of the latent virus, giving rise to a productive infection and allowing HCMV to disseminate into host tissues [22, 25, 31, 33–35]. On that basis, a number of experimental models have been developed in order to try to reproduce the in vivo events occurring upon HCMV infection. Among the in vitro cell lines mimicking the natural system, human monocytic leukemia cells (THP-1) are widely used as a model of HCMV latent infection [36–40]. These cells are non-permissive to the lytic cycle, while harbouring the viral genome [37, 39, 41]. THP-1 monocytes have also largely been employed as a HCMV lytic infection model; to this end, they are first differentiated into macrophages by treatment with a phorbol ester before HCMV infection in order to render the cells permissive to HCMV infection [33, 37, 42–44].

On the other hand, the use of THP-1 cells as a HCMV reactivation model (by inducing differentiation into macrophages after infection) has never been clearly demonstrated. Some studies have provided evidence of HCMV reactivation by looking at the viral immediate-early (IE) genes expression alone [45], whereas others have looked also at early gene products (DNA polymerase or other accessory proteins) [46, 47], or even at a late protein [48]. To demonstrate completion of the lytic cycle by the demonstration of HCMV progeny production, the co-cultivation of infected THP-1 cells with human fibroblasts has been used [49]. However, these fragmentary approaches have given rise to doubts concerning the use of THP-1 monocytes as a true latency-reactivation model [40].

The aim of the present study was to investigate the effectiveness of THP-1 cells as a good latency-reactivation model by analysing all the steps of HCMV lytic cycle, giving rise to a viral yield upon reactivation, in order to entirely reproduce the features of viral infection that occurs in vivo upon differentiation of monocytes into macrophages, i.e. one of the cell types responsible for HCMV dissemination into host tissues.

To this end, following HCMV TB40E infection at low multiplicities of infection and subsequent induction of THP-1 differentiation into macrophages, we monitored the development and completion of the HCMV lytic cycle by analysing the viral DNA amounts in both cell models, the whole pattern of transcripts representative of the HCMV lytic programme, and the production of infectious progeny by directly analysing the culture medium of infected THP-1 cells; the viral *inoculum* was also checked for the presence of exosomes.

Viral yield production from THP-1 latency and reactivation models was also analysed using the highly laboratory passaged Towne strain.

Our results clearly demonstrate that THP-1 cells constitute a true HCMV reactivation model from latency and provide an effective tool for studies aimed at elucidating the mechanisms that regulate the switch between latency and reactivation.

## Methods

### Cell culture

The THP-1 monocytic cell line (“Istituto Zooprofilattico Sperimentale della Lombardia e dell’Emilia-Romagna”) was maintained in suspension in Roswell Park Memorial Institute (RPMI) 1640 medium, supplemented with 1 % L-glutamine, 1 % sodium pyruvate, 50 μM β-mercaptoethanol, 10 % foetal bovine serum (FBS) and antibiotics (100 U/ml penicillin, 100 μg/ml streptomycin). For infection experiments, THP-1 cells were seeded into 6-well plates to produce a final density of 1.7 × 10^6^ cells/ml. Cell differentiation into macrophages was induced by adding 80 nM 12-*O*-tetradecanoylphorbol-13-acetate (TPA, Sigma-Aldrich) to the medium. TPA treatment resulted in cell adhesion to the substrate, the presence of macrophage differentiation markers and phenotype modification (large flattened cells). Adherent cell counting (20 randomly selected fields) and flow cytometry analysis (see below) were performed to check the differentiation efficiency.

Monolayer cultures of MRC5 human embryo lung fibroblasts (American Type Culture Collection, ATCC; CCL-171) were grown in Earle’s modified Minimum Essential Medium (E-MEM), supplemented with 1 % L-glutamine, 1 % non-essential amino acids, 1 mM sodium pyruvate, 10 % FBS and antibiotics (100 U/ml penicillin, 100 μg/ml streptomycin). Cell culture medium and supplements were from Euroclone.

### Virus strain and titration

HCMV TB40E reference strain (kindly provided by Prof. Thomas Mertens, Institute of Virology, Ulm University, Germany) and Towne strain (ATCC VR-977) were propagated in MRC5 cells; the viral infectious titre was determined as previously described [50].

### Virus inactivation

For the UV inactivation of HCMV TB40E, an aliquot of virus was diluted into 1 ml of RPMI without FBS (to obtain an MOI of 0.5 for the indicated infection times and conditions and transferred to a 3 cm Petri dish. The viral suspension was placed on ice and UV-irradiated as previously described [42].

### Viral infection of latency, reactivation and lytic cell models

Infection experiments in non-differentiated THP-1 monocytes (latency model) were performed as follows: cells were first centrifuged at low speed for 10 min (500 × *g*), then the cell pellet was gently suspended in viral-enriched culture medium at three different multiplicities of infection (MOI): 0.5, 0.25 and 0.125 plaque forming units (PFU)/cell. THP-1 cells were then seeded into 6-well plates (final density of 1.7 × 10^6^ cells/ml) and centrifuged at 700 × *g* for 45 min. Finally, the cells were incubated at 37 °C for 75 min. After the absorption period, the virus *inoculum* was removed and replaced with RPMI supplemented with 10 ﻿% FBS. The infected cells were incubated at 37 °C for the indicated time periods.

For the reactivation model, after one day-incubation at 37 °C, aliquots of THP-1 latently infected monocytes were differentiated into macrophages by adding 80 nM TPA to the medium for 48 h; then, TPA-supplemented medium was gently removed and replaced by fresh medium. Differentiated cells were incubated at 37 °C for 30 h, 4, 6 or 7 days p.i.. Adherent cell counting was performed as mentioned above for uninfected cells.

For the lytic model, THP-1 monocytes were differentiated into macrophages by adding 80 nM TPA to the medium for 48 h. The medium was then removed and the adherent cells infected with HCMV at an MOI 0.5, 0.25 or 0.125. Virus adsorption was performed by centrifugation at 700 × *g* for 45 min. Cells were then incubated at 37 °C for 75 min. Subsequently, the virus *inoculum* was removed and replaced with RPMI supplemented with 10 % FBS. The infected cells were incubated at 37 °C for the indicated times.

As for HCMV infection of MRC5 fibroblasts, virus adsorption was performed by centrifugation at 700 × *g* for 45 min followed by a 15 min-incubation at 37 °C; E-MEM supplemented with 10 % FBS was then added after removing the viral *inoculum*.

### Cell fractionation

Cell fractionation was performed as previously described [51]. Briefly, at 30 h, 4, 6 and 7 days p.i. non-differentiated and differentiated THP-1 cells (the latter being adherent, thus were first dissociated by trypsinization) were collected and centrifuged at 500 × *g* for 10 min. Cell pellets were washed twice with phosphate-buffered saline (PBS, pH 7.4: 7 mM Na_2_HPO_4_, 1.5 mM KH_2_PO_4_, 137 mM KCl) by centrifugation at 500 × *g* for 10 min. Before fractionation, cell aliquots were counted in the presence of the vital stain Trypan blue (Sigma-Aldrich) in order to assess the percentage of viable cells. All the following steps were performed at 4 °C. Cells were gently resuspended in 8 volumes of hypotonic buffer (10 mM triethanolamine, pH 7.4, 10 mM KCl, 1 mM MgCl_2_, 1 mM MnCl_2_, 5 mM 2-mercaptoethanol) and homogenized in a tight Dounce homogenizer. Lysis was monitored by a phase-contrast microscope and isotonicity was restored after 5–6 min by addition of sucrose 2 M (0.25 M final). Nuclei were collected by centrifugation at 1200 × *g* for 10 min and freed of residual cytoplasmic material in a glass Dounce homogenizer by resuspension with a loose pestle in 8 volumes of isotonic buffer (hypotonic buffer additioned with 0.25 M sucrose) containing 0.3 % NP40 as previously described [52].

The supernatant, corresponding to the cytoplasmic fraction, was harvested and stored at −20 °C for further analysis. Nuclei were resuspended in isotonic buffer and sonicated using a HD 2070 Bandelin Sonopuls Ultrasonic homogenizer on ice for three bursts of 6 s each, separated by 6 s intervals. The obtained nuclear fractions were used for DNA extraction.

Cellular fractions (total cellular lysate, nuclear and cytoplasmic fractions) were also subjected to protein quantification [53]. All chemicals were from Sigma-Aldrich.

### Exosome extraction

Exosomes were purified after 7 days of infection with TB40E strain at an MOI 0.5 from cell culture supernatants of infected THP-1 derived-macrophages (reactivation models) and uninfected THP-1 derived-macrophages (1.7 × 10^6^ cells/ml).

Cell culture supernatants were sequentially centrifuged: at 1000 × *g* for 10 min to remove floating cells, at 10,000 × *g* for 10 min to remove smaller cell debris and then were ultracentrifuged at 100,000 × *g* for 90 min for exosomes collection. The final pellet was resuspended in PBS and passed through a 0.2 μM filter. All procedures were carried out at 4 °C [54]. The obtained protein suspensions were subjected to quantification [53].

### Protein precipitation

According to protein quantification, different volumes of each of the cellular fractions (total cell lysate, nucleus and cytoplasm) from both the latency and reactivation cell models were subjected to trichloroacetic acid (TCA, Sigma-Aldrich) precipitation to obtain equal amounts of proteins; TCA (1:10 v/v) was added to each protein suspension and incubated on ice for 30 min after mixing. Proteins were precipitated by centrifugation at 12,000 × *g* for 20 min at 4 °C and the supernatants were discarded. The pellets were resuspended in 1 ml ice-cold acetone (Carlo Erba) and incubated on ice for 10 min. Then, they were centrifuged at 12,000 × *g* for 15 min at 4 °C and air-dried at room temperature for 5 min. Subsequently, they were dissolved in 15 μl Laemmli buffer. Tris-HCl (1 M, pH 8.5) was used to adjust the pH to 6.8. Equal amounts (30 μg) of each fraction were analyzed by 12.5 % (or 10 % for exosomes) sodium dodecyl sulfate-polyacrylamide gel electrophoresis (SDS-PAGE).

### Western blot (WB) analysis

Proteins resolved by SDS-PAGE were transferred to a nitrocellulose membrane. After a blocking step in non-fat dry milk for 30 min at room temperature, the membranes were incubated for 1 h and 30 min in non-fat dry milk with two primary antibodies added simultaneously: rabbit polyclonal anti-nucleophosmin (B23, Santa Cruz Biotechnology; 1:100 dilution) and mouse monoclonal anti-beta-actin (Biovision; 1:1000 dilution). For exosome analysis, a monoclonal antibody anti-Alix (HansaBiomed; 1:500 dilution) was employed. As positive control, a lyophilised exosome standard derived from the cell culture medium of COLO1 cells (HansaBiomed) was used. After four washing steps in PBS supplemented with 0.2 % Tween20 (Sigma-Aldrich), the membranes were incubated for 1 h in PBS containing alkaline phosphatase (AP)-conjugated anti-rabbit (Santa Cruz Biotechnology; 1:600 dilution) and anti-mouse (Sigma Aldrich; 1:6000 dilution) antibodies. Then, the membranes were re-washed as described above, and the immunoreaction was visualised using Sigma Fast BCIP/NBT-buffered substrate (Sigma Aldrich). Molecular weight (MW) markers were from Novex® Sharp (3.5–260 kDa Pre-Stained Protein Standard) or from PanReac AppliChem (6.5–200 kDa prestained Protein Marker II) in the case of exosome analysis.

### DNA extraction and q-Real-time PCR assay

Total DNA was extracted from the nuclear fractions using the NucliSENS® EasyMAG® platform (bioMérieux). The obtained DNA was subjected to q-Real-Time PCR amplification using the CMV ELITe MGB® Kit (ELITechGroup) for the detection and quantification of the human HCMV DNA exon 4 region of the immediate-early (IE)1 gene. The cellular beta-globin gene was qualitatively co-amplified. The assay was performed according to the manufacturer’s instructions using the 7300 Real-time PCR system (ABI PRISM, Applied BioSystems). The results were expressed as DNA copies/ml (logarithmic scale).

### RNA extraction and reverse transcription (RT)-PCR

Total RNA was extracted from non-differentiated infected cells (latency model) or infected and differentiated cells (reactivation model) at 7 days p.i. using the Nucleo Spin® RNA II kit according to the manufacturer’s instructions (Macherey-Nagel). Template RNAs were then reverse transcribed using the RevertAid First Strand cDNA Synthesis Kit (Thermo Scientific). The obtained cDNA was subjected to PCR amplification using specific primers for the anti-sense cytomegalovirus latency-associated transcript (anti-sense CLT, amplification product: 469 bp) [47] and for viral immediate-early (IE1, amplification product: 303 bp), early (DNA pol, amplification product: 237 bp), early-late (pp65, amplification product: 213 bp) and late (pp150, amplification product: 206 bp) lytic transcripts [37]. The thermal cycling conditions for the anti-sense CLT transcript were: 2 min of denaturation at 94 °C followed by 50 cycles of 5 s at 94 °C and 30 s at 70 °C. The thermal cycling conditions were similar for all HCMV lytic transcripts: 2 min denaturation at 94 °C followed by 35 cycles of 30 s at 94 °C, 30 s at the specific annealing temperatures (55 °C, 56 °C, 57 °C, 59 °C, for DNA pol, IE1, pp150 and pp65 respectively) and 30 s at 72 °C [37]. The glyceraldehyde-3-phosphate-dehydrogenase (GAPDH) cellular transcript fragment of 242 bp was amplified as a reaction control. To confirm the absence of viral DNA contamination, PCR was also performed on non-retrotranscribed RNA extracts for each of the above mentioned viral products. PCR products were analyzed by electrophoresis on a 1 % agarose gel using ethidium bromide staining. Gel electrophoresis images were acquired using the Gel Doc EZ system (Bio-Rad).

### Viral progeny analysis by indirect immunofluorescence assay and titration of viral antigen-positive cells

Analysis of the viral yield obtained from the THP-1 latency, reactivation and lytic models, as well as the evaluation of the progeny obtained by directly infecting MRC5 fibroblasts, was first performed as briefly described hereafter. Following the infection of THP-1 monocytes, THP-1 derived-macrophages (differentiated after or before infection to obtain reactivation and lytic models, respectively) and MRC5 fibroblasts all infected at an MOI 0.5, 0.25 or 0.125 for 7 days, the culture medium was collected and centrifuged at 13,900 × *g* for 30 min at 4 °C then ultracentrifuged at 60,000 × *g* for 1 h at 4 °C.

Each pellet was resuspended in 200 μl E-MEM without FBS, and the whole volume used to infect MRC5 fibroblasts grown as monolayers on shell vials (final cell concentration: 5.4 × 10^5^/shell vial). Virus adsorption was performed by centrifugation at 700 × *g* for 45 min followed by a 15 min-incubation at 37 °C; E-MEM supplemented with 10 % FBS was then added after removing the viral *inoculum*.

After a 24 h- (for IE detection) or 48 h-infection (for pp65 detection), MRC5 infected cells grown on shell vials were gently fixed with methanol at −20 °C for 10 min and then stained and analyzed as previously described [50]. Alternatively, THP-1 macrophages (lytic model) grown on shell vials (9 × 10^5^/shell vial) were used for the above mentioned analyses.

A purified monoclonal antibody (Mab clone E13; working dilution 1:20) specific for a common epitope encoded by the exon 2 of the major IE1 and IE2 genes (Argène) and a purified monoclonal antibody (clones 1C3 and AYM-1; working dilution 1:20) reacting with the 65–68 kDa HCMV lower matrix structural phosphoprotein pp65 (Argène) were used as primary antibodies. Cells were incubated at 37 °C for 1 h and then washed three times with PBS 1X. The immunoreactions were revealed by Alexa-fluor fluorescein isothiocyanate (FITC)-conjugated goat anti-mouse IgG (Argène; working dilution 1:500). The fluorescent DNA dye 4′,6-diamidino-2-phenylindole (DAPI) and Evans blue were used as secondary antibody diluents to counterstain nuclear chromatin (blue) and cells (red), respectively. Finally, cells were mounted with Prolong Gold anti-fade reagent (Molecular Probes) and analyzed by fluorescence microscopy (Leica DMLB).

To perform a quantitative evaluation of IE- and pp65-positive MRC5 fibroblasts, ten randomly selected fields per slide were counted and IE- or pp65-positive cells expressed as mean percentage values of the total cell number per field (280 to 340 total MRC5 fibroblasts evaluated by DAPI counterstaining of nuclei).

### THP-1- and MRC5-derived viral yield titration by 50 % percent Tissue Culture Infectious Dose (TCID_50_) assay

Ten-fold serial dilutions of culture supernatants from infected cells (THP-1 reactivation and lytic models, MRC5 fibroblasts) were prepared in FBS-free E-MEM. To this end, 100 μl of diluted supernatants were added to each well of a 96-well microplate (4 replicates per dilution), then 100 μl of a freshly prepared MRC5 cellular suspension (cell density: 6 × 10^5^ cells/ml in E-MEM supplemented with 5 % decomplemented FBS) were overloaded. Viral yields were evaluated after 5–6 days of incubation at 37 °C. The TCID_50_ values were calculated using the method established by Reed and Muench [55].and expressed as TCID_50_ values per 0.1 ml of culture supernatant.

### Flow cytometry

THP-1 uninfected monocytes and monocytes-derived macrophages were processed as follows.

THP-1 monocytes were seeded in a 6-well plate at a concentration of 1.7 × 10^6^ cells/ml. Aliquots were then differentiated into macrophages as described in the appropriate section above. THP-1 macrophages were trypsinized at 4 and 6 days after differentiation. To analyse the presence of the surface differentiation CD11b and CD14 markers by flow cytometry, aliquots of 0.5 × 10^6^ cells per experimental point were subjected to single staining by using FITC-conjugated monoclonal antibodies to CD11b and CD14 antigens (Becton Dickinson). Non-specific fluorescence was assessed by using isotype-matched controls. Data collected from 10,000 cells are reported as either percentages of positive THP-1 monocytes or mean fluorescence intensity (MFI) values. The analyses were performed using an EPICS® XL-MCL flow cytometer and Expo32ADC Software (Beckman Coulter).

## Results

In a preliminary series of experiments we wanted to ascertain the validity of our experimental model.

We first investigated the expression of CD11b and CD14 differentiation markers in uninfected THP-1 monocytes vs macrophages by flow cytometry at 4 and 7 days after TPA stimulation (Additional file 1). Concerning CD14, the results demonstrate that it is already highly expressed in THP-1 monocytes; thus its expression does not significantly increase upon TPA stimulation in terms of number of cells (panel A), in agreement with other data in the literature [56, 57]. Nevertheless, the evaluation of the Mean Fluorescence Intensity (MFI) which reflects the concentration of fluorescent conjugates and the receptors they stain, shows that CD14 expression intensity increases in THP-1 macrophages (panel B). As for CD11b, the number of cells expressing this receptor was found to be significantly higher in THP-1 macrophages (panel A), while MFI expression was shown to be low and quite constant in both cell types (panel B).

In order to assess the differentiation rate of the reactivation model, we counted the adherent cells of 20 randomly selected fields of uninfected monocytes differentiated into macrophages vs latently infected monocytes differentiated after infection. The two conditions show quite comparable results (94 % of uninfected monocytes-derived THP-1 macrophages vs 85 % adherent cells in the reactivation model), although those in the latter model were slightly lower. This could be explained by the need to apply the experimental procedure necessary for virus infection, which includes a centrifugation step, compatible with an acceptable cell loss. The few residual floating cells were discarded by renewing the maintenance medium after a 48 h-TPA differentiation. These cells were not characterized as the scope of this study was to assess the infection efficiency of the reactivation model.

### HCMV DNA levels in the latent and reactivation models after 30 h, 4, 6 and 7 days of infection

HCMV infection was carried out using a range of low MOI (0.5, 0.25, 0.125). In order to evaluate the amounts of virus that had been internalized and relocated to the nucleus, the presence of viral DNA was only analyzed in the nuclear fractions of infected THP-1 cells. This allowed us to exclude the viral particles that may remain adherent to the cytoplasmic membrane without giving rise to infection. Nuclei fractions derived from the latency and reactivation models (HCMV-infected THP-1 monocytes and THP-1 monocytes differentiated into macrophages after infection, respectively) were obtained by cellular fractionation. Cell counting was performed in the presence of the vital stain Trypan blue before fractionation. The percentage of viable cells for each MOI at the time points considered for viral DNA evaluation were comparable between the latency and reactivation models (92 to 76 % for the latent model vs 89 to 70 % for the reactivation model from 30 h to 7 days).

**Fig. 1:**
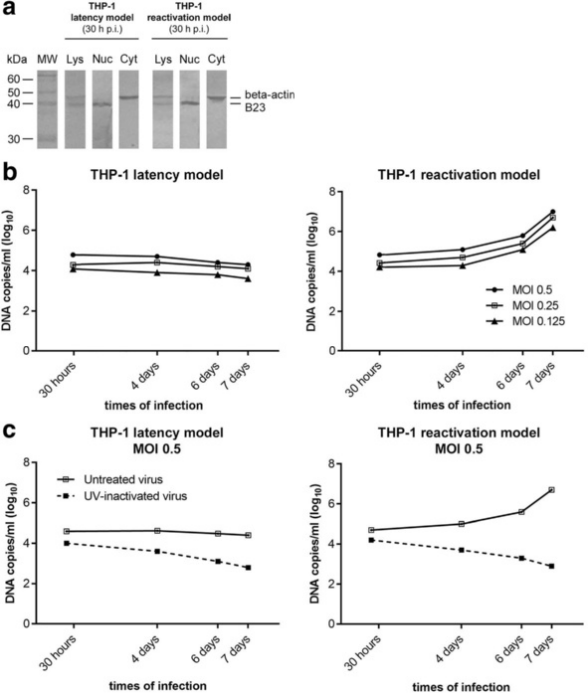
Time-course changes in HCMV DNA nuclear amounts in THP-1 latency and reactivation models. **a** At the indicated time points p.i., undifferentiated (panel “THP-1 latency model”) and differentiated THP-1 cells (panel “THP-1 reactivation model”) were subjected to cellular fractionation in order to obtain purified nuclei (Nuc) and cytoplasmic (Cyt) fractions derived from total cell lysates (Lys). After SDS-PAGE, WB analysis was done using a rabbit polyclonal antibody directed to B23 (40 kDa) and a mouse monoclonal anti-beta-actin (44 kDa) antibody as nuclear and cytoplasmic markers, respectively. The immunoreactions were revealed by AP-conjugated anti-rabbit and anti-mouse antibodies. Molecular weight markers are indicated on the left-hand figure. **b** Total DNA was extracted from nuclei of undifferentiated THP-1 monocytes (panel “THP-1 latency model”) and THP-1 monocytes differentiated after infection (panel “THP-1 reactivation model”). HCMV DNA amounts were measured by q-Real-time PCR at 30 h, 4, 6 and 7 days p.i. at MOI 0.5, 0.25 and 0.125 in both experimental models. Comparable results were obtained from two independent experiments. **c** Total DNA was extracted from the nuclei of “viable” HCMV-infected THP-1 monocytes (“untreated virus”) and UV-inactivated HCMV-infected THP-1 monocytes (panel “THP-1 latency model, MOI 0.5”) and THP-1 monocytes differentiated after infection (panel “THP-1 reactivation model, MOI 0.5”). HCMV DNA amounts were measured by q-Real-time PCR at 30 h, 4, 6 and 7 days p.i. at MOI 0.25 in both experimental models

Cell fractionation was preliminarily validated by WB analysis, demonstrating the correct location of nuclear and cytoplasmic cell markers (Fig. 1a: “THP-1 latency model 30 h p.i.” and “THP-1 reactivation model 30 h p.i.”).

The presence and quantification of HCMV DNA from the cell nuclei of the latency and reactivation models was first assessed by Real-time PCR at 30 h p.i. for the three MOI used (0.5, 0.25, 0.125) (Fig. 1b, panels “THP-1 latency model” and “THP-1 reactivation model”). At this time p.i., the results obtained in the latency and the reactivation models were comparable, with decreasing HCMV DNA copies/ml compatible with the decreasing MOI used in the experimental models.

We next focused our attention on the Real-time PCR results obtained by amplifying the DNA extracted from the nuclear fractions obtained from the two HCMV infection models after 4, 6 and 7 days of infection at the three considered MOI. In the latency model, the HCMV DNA copies/ml obtained after 4, 6 and 7 days of infection were comparable to the value obtained after just 30 h of infection for each of the considered MOI (Fig. 1b, panel “THP-1 latency model”), although a small decrease could be noted in viral DNA amounts, becoming most evident on day 7. In contrast, in the reactivation model, HCMV DNA copies/ml gradually increased from 4 to 7 days of infection for all the considered MOI (Fig. 1b, panel “THP-1 reactivation model”). The values obtained in two independent experiments and the related standard deviations are shown in Table 1.

**Table 1:** Viral DNA content in THP-1 latency and reactivation models (two independent experiments) and standard deviations

Times of infection	MOI (PFU/cell)	THP-1 latency model					THP-1 reactivation model				
		DNA copies/ml		DNA copie/ml (log_10_)		SD^a^ (log_10_)	DNA copies/ml		DNA copie/ml (log_10_)		SD^a^ (log_10_)
		1	2	1	2		1	2	1	2	
30 h	0.125	1.32 × 10^4^	1.29 × 10^4^	4.121	4.111	0.007	1.44 × 10^4^	1.95 × 10^4^	4.158	4.290	0.094
	0.25	2.04 × 10^4^	2.21 × 10^4^	4.309	4.344	0.024	2.13 × 10^4^	2.69 × 10^4^	4.328	4.430	0.072
	0.5	6.51 × 10^4^	6.80 × 10^4^	4.813	4.832	0.013	5.80 × 10^4^	6.09 × 10^4^	4.763	4.785	0.015
4 days	0.125	8.96 × 10^3^	8.12 × 10^3^	3.952	3.909	0.030	2.44 × 10^4^	2.30 × 10^4^	4.387	4.362	0.018
	0.25	2.81 × 10^4^	2.91 × 10^4^	4.449	4.464	0.010	5.56 × 10^4^	5.80 × 10^4^	4.745	4.763	0.013
	0.5	4.71 × 10^4^	4.95 × 10^4^	4.673	4.695	0.015	1.32 × 10^5^	1.54 × 10^5^	5.120	5.187	0.046
6 days	0.125	6.31 × 10^3^	6.91 × 10^3^	3.800	3.839	0.028	1.53 × 10^5^	1.27 × 10^5^	5.185	5.104	0.056
	0.25	1.89 × 10^4^	1.75 × 10^4^	4.277	4.243	0.023	2.97 × 10^5^	2.50 × 10^5^	5.473	5.398	0.053
	0.5	2.75 × 10^4^	2.61 × 10^4^	4.440	4.417	0.016	7.30 × 10^5^	6.50 × 10^5^	5.863	5.813	0.035
7 days	0.125	4.80 × 10^3^	4.21 × 10^3^	3.681	3.624	0.040	1.32 × 10^6^	1.70 × 10^6^	6.120	6.230	0.077
	0.25	1.50 × 10^4^	1.30 × 10^4^	4.176	4.113	0.044	5.58 × 10^6^	5.42 × 10^6^	6.747	6.734	0.009
	0.5	2.03 × 10^4^	2.14 × 10^4^	4.307	4.330	0.016	9.08 × 10^6^	1.02 × 10^7^	6.958	7.006	0.034

^a^ *SD* standard deviation

These experiments were repeated using UV-inactivated virus at an MOI of 0.5 (Fig. 1, panel c). The results show that the UV-inactivated virus DNA enters the cell nuclei in both models, and that DNA levels gradually decrease throughout the considered infection times.

The applied Real-time method also included the qualitative co-amplification of the THP-1 beta-globin gene. In our experiments, its threshold cycle value was 26 for the time points tested in the latent model and 25 in the reactivation model.

### HCMV transcript analysis

**Fig. 2:**
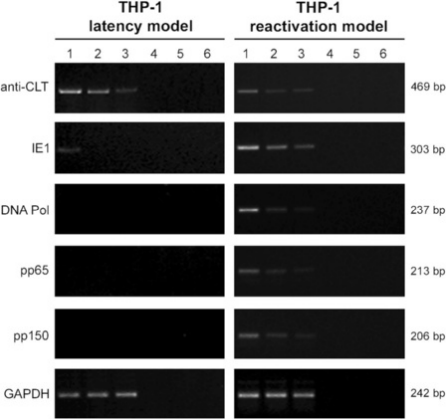
Analysis of HCMV transcripts from THP-1 latency and reactivation models at 7 days p.i.. The expression patterns of the anti-sense CLT (cytomegalovirus latency associated transcript), IE1 (immediate-early), DNA pol (early), pp65 (early-late) and pp150 (late) viral transcripts were analysed in both models (panels “THP-1 latency model” and “THP-1 reactivation model”) after infection at three different MOI. The cellular GAPDH housekeeping gene amplification product was used as a reaction control. Lanes 1, 2 and 3: MOI 0.5, 0.25, 0.125, respectively; lanes 4, 5 and 6: DNA contamination controls (PCR performed for MOI 0.5, 0.25, 0.125, respectively, in the absence of reverse transcription). Molecular weights of the amplified transcript fragments are shown on the right side. The results are representative of two independent experiments

To verify the triggering and the development of the HCMV lytic programme in THP-1 monocytes differentiated into macrophages after infection (reactivation model), we analysed the presence of viral transcripts representative of the immediate-early, early, early-late and late phases of the HCMV lytic cycle (IE1, DNA pol, pp65 and pp150, respectively) at 7 days p.i. (Fig. 2, panel “THP-1 reactivation model”).

In order to ascertain whether the presence of viral transcripts was the result of HCMV reactivation induced by THP-1 monocyte differentiation, RT-PCR experiments were also performed using the latency model (Fig. 2, panel “THP-1 latency model”).

RT-PCR products for HCMV IE1, DNA pol, pp65 and pp150 transcripts were found in the reactivation model; for each of the considered transcripts, the signal intensity decreased according to the decreasing MOI used (Fig. 2, panel “THP-1 reactivation model”, lane 1: MOI 0.5; lane 2: MOI 0.25; lane 3: MOI 0.125; lanes 4, 5 and 6 were non retro-transcribed controls, as detailed in the figure legend). Conversely, no amplification products could be detected in the latency model, with the exception of a very faint signal for the IE1 transcript at an MOI of 0.5 (Fig. 2, “THP-1 latency model”, lane 1).

Furthermore, we demonstrated the presence of the anti-sense cytomegalovirus latency-associated transcript in the latency model for all the considered MOI (Fig. 2, panel “THP-1 latency model”); fainter anti-sense cytomegalovirus latency-associated transcript signals were also detected in the reactivation model (Fig. 2, panel “THP-1 reactivation model”). The cellular GAPDH housekeeping gene amplification product was used as reaction control.

### THP-1-derived viral progeny analysis

**Fig. 3:**
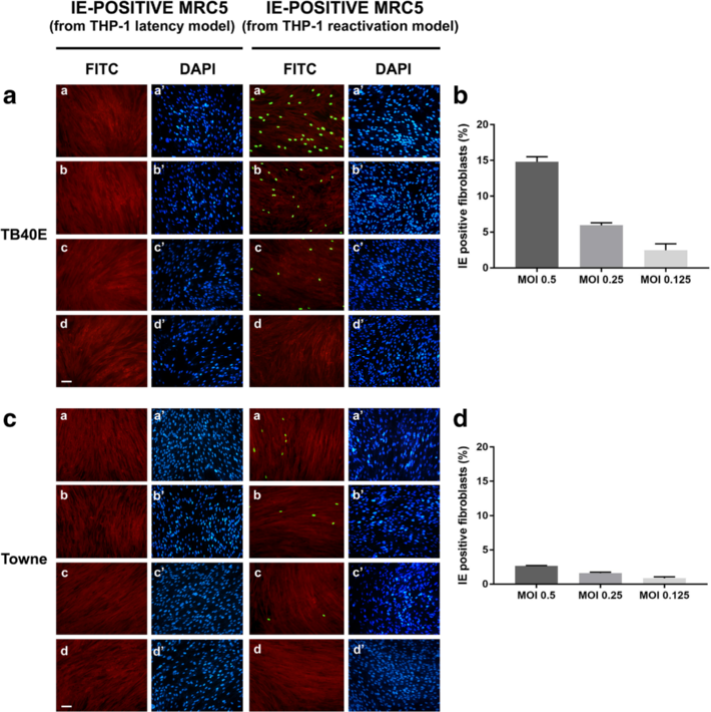
Analysis of HCMV progeny from THP-1 latency and reactivation models: IE antigen immunofluorescence pattern. **a** and **c** MRC5 fibroblasts were infected with the cell culture medium derived from THP-1 monocytes (“IE-positive MRC5 from THP-1 latency model”) and THP-1 macrophages (“IE-positive MRC5 from THP-1 reactivation model”) which had been infected with TB40E (**a**) or Towne (**c**) HCMV strains at MOI of 0.5 (panels a, a’), 0.25 (panels *b*, *b’*) or 0.125 (panels *c*, *c’*) for 7 days. At 24 h p.i., MRC5 cells were fixed and labelled with a monoclonal antibody specific for the common epitope encoded by exon 2 of HCMV IE1 and IE2 genes. The immunoreaction was revealed by Alexa-Fluor FITC-conjugated goat anti-mouse IgG (panels *a*, *b*, *c*, *d*: FITC, green nuclei). Cells were counterstained with Evans blue (panels *a*, *b*, *c*, *d*: red cells) and DAPI (panels *a’*, *b’*, *c’*, *d’*: blue nuclei). Panels *d*, *d’*: uninfected cells. Images were collected using a conventional fluorescence microscopy. Bar: 25 μm. **b** and **d** The quantitative evaluation of IE-positive MRC5 fibroblasts infected with the cell culture medium derived from TB40E (**b**) or Towne (**d**) reactivation models. Values were expressed as mean percentages of IE-positive cells per field (ten randomly selected fields per slide were counted) from two independent experiments; error bars indicate standard deviations. Values were processed using GraphPad Prism 7 software

**Fig. 4:**
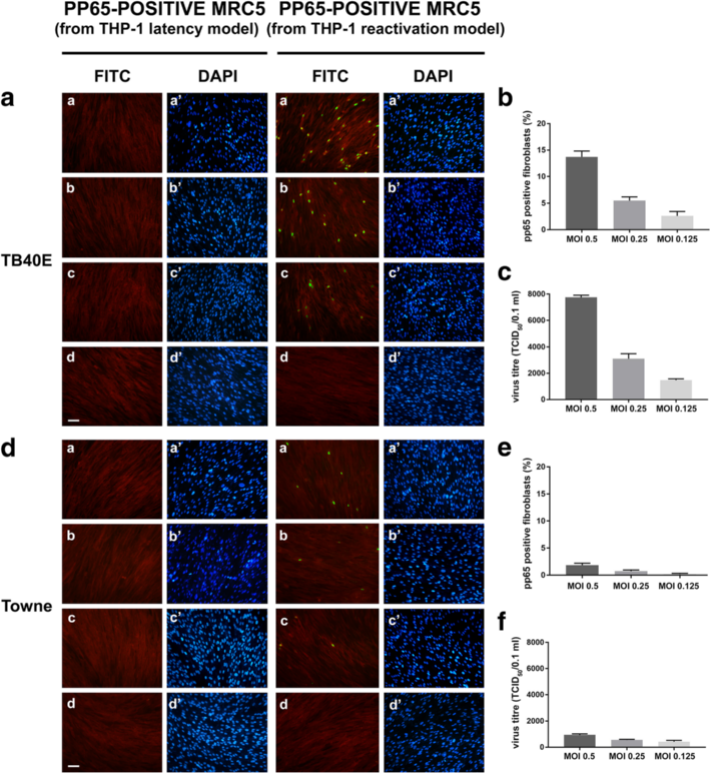
Analysis of HCMV progeny from THP-1 latency and reactivation models: pp65 antigen immunofluorescence pattern and viral yield titration. **a** and **d** MRC5 fibroblasts were infected with cell culture medium derived from THP-1 monocytes (“pp65-positive MRC5 from THP-1 latency model”) and THP-1 macrophages (“pp65-positive MRC5 from THP-1 reactivation model”) which had been infected with TB40E (**a**) or Towne (**d**) HCMV strains at MOI of 0.5 (panels *a, a’*), 0.25 (panels b, b’) and 0.125 (panels *c, c’*) for 7 days. At 48 h p.i., MRC5 cells were fixed and labelled with a monoclonal antibody reacting with the viral matrix phosphoprotein pp65. The immunoreaction was revealed by Alexa-Fluor FITC-conjugated goat anti-mouse IgG (panels *a, b, c, d*: FITC, green nuclei). Cells were counterstained with Evans blue (panels *a, b, c, d*: red cells) and DAPI (panels *a’, b’, c’, d’*: blue nuclei). Panels *d, d’*: uninfected cells. Images were collected using a conventional fluorescence microscopy. Bar: 25 μm. **b** and **e** The quantitative evaluation of pp65-positive MRC5 fibroblasts infected with the cell culture medium derived from TB40E (**b**) or Towne (**e**) reactivation models. Values were expressed as mean percentages of pp65-positive cells per field (ten randomly selected fields per slide were counted) from two independent experiments; error bars indicate standard deviations. Values were processed using GraphPad Prism 7 software. **c** and **f** Virus yields were evaluated by the TCID_50_ assay from cell culture medium derived from TB40E (**c**) or Towne (**f**) reactivation models, as detailed in the “Methods” section. Two independent experiments were performed; error bars in graphs represent standard deviations. Values were processed by the GraphPad Prism 7 software

To verify the completion of the TB40E strain replication cycle, we evaluated the viral progeny production in the reactivation model at 7 days p.i.. The same experimental procedure described below was also applied to the latency model as a negative control; furthermore, the efficiency of TB40E productive replication upon reactivation was compared with that obtained using the laboratory passaged Towne strain (Figs. 3 and 4). To this end, the culture supernatants obtained from TB40E or Towne latency and reactivation models at 7 days p.i. were processed as detailed in the Methods section and used for the infection of MRC5 human fibroblasts (a cell model highly permissive to lytic HCMV infection in vitro). IE-positive cells were checked by the detection of IE HCMV antigens in MRC5 infected nuclei by immunofluorescence, using antibodies directed against a common epitope of IEp72 and IEp86 viral proteins (Fig. 3a: TB40E; Fig. 3c: Towne; a, b, c and a’, b’, c’ panels show the results for MOI 0.5, 0.25. 0.125, respectively). The quantitative evaluation of infected (IE-positive) MRC5 fibroblasts is shown in Fig. 3b, d for TB40E and Towne, respectively. Similarly, the presence and amount of the late pp65 viral antigen was checked by immunofluorescence following the infection of MRC5 fibroblasts with the supernatants derived from TB40E or Towne latency or reactivation models (Fig. 4a, b: TB40E; Fig. 4 d, e: Towne).

The TB40E and Towne yields obtained upon reactivation were titrated using the TCID_50_ assay, as detailed in the “Methods” section (Fig. 4c, f for TB40E and Towne, respectively). The results are consistent with the presence of an infectious viral yield in the reactivation model and its absence in the latency model for both viruses; the infection efficiency of the TB40E endotheliotropic strain was shown to be higher that than of the Towne strain.

The viral progeny obtained upon TB40E reactivation was also tested for its ability to infect macrophagic-like cells, like the THP-1 differentiated before infection. The expression of IE and pp65 antigens was monitored by immunofluorescence and the results are shown in Additional file 2. As expected, the observed infection efficiency was lower than that obtained using MRC5 fibroblasts (compare Additional file 2A to Fig. 3a and Additional file 2B to Fig. 4a).

**Fig. 5:**
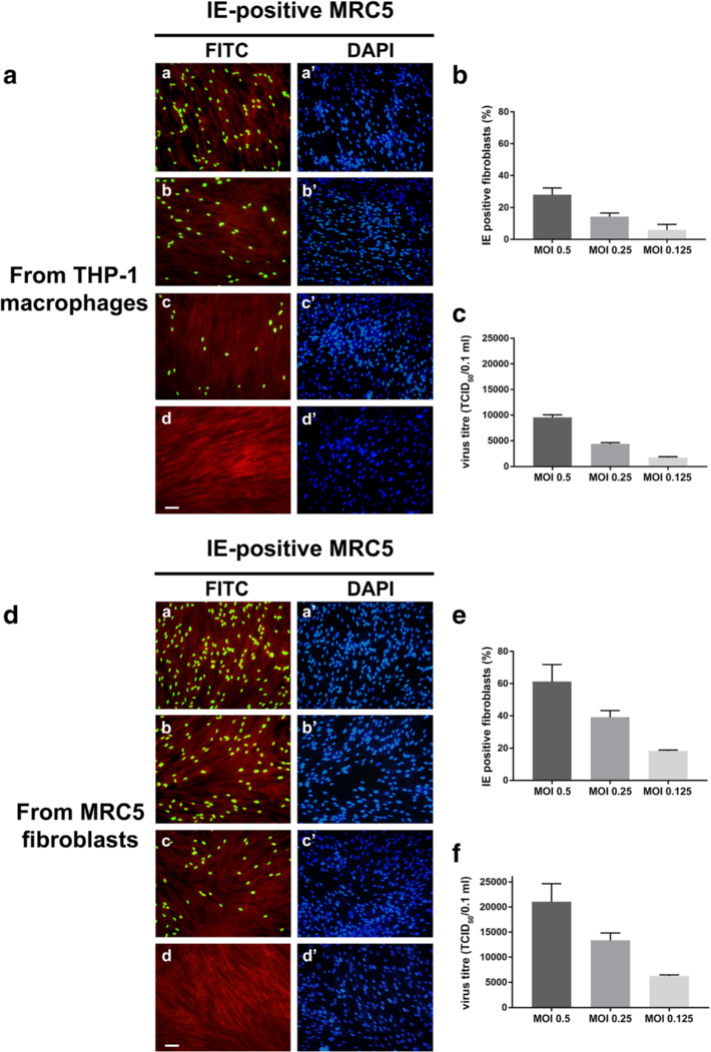
Analysis of TB40E progeny from THP-1 macrophages (lytic model), and MRC5 fibroblasts. **a** and **d** MRC5 fibroblasts were infected with cell culture medium derived from THP-1 lytic model (**a**) or from MRC5 fibroblasts (**d**) which had been infected with HCMV TB40E at MOI of 0.5 (panels *a, a’*), 0.25 (panels *b, b’*) and 0.125 (panels *c, c’*) for 7 days. At 24 h p.i., MRC5 cells were fixed and labelled with a monoclonal antibody specific for the common epitope encoded by exon 2 of HCMV IE1 and IE2 genes. The immunoreaction was revealed by Alexa-Fluor FITC-conjugated goat anti-mouse IgG (panels *a, b, c, d*: FITC, green nuclei). Cells were counterstained with Evans blue (panels *a, b, c, d*: red cells) and DAPI (panels *a’, b’, c’, d’*: blue nuclei). Panels *d, d’*: uninfected cells. Images were collected using a conventional fluorescence microscopy. Bar: 25 μm. **b** and **e** The quantitative evaluation of IE-positive MRC5 fibroblasts infected with the cell culture medium derived from THP-1 lytic model (**b**) or MRC5 fibroblasts as already described (see Fig. 3 legend) (**e**). **c** and **f** Virus yields were evaluated by the TCID_50_ assay from cell culture medium derived from THP-1 lytic model (**c**) or MRC5 fibroblasts (**f**), as detailed in the “Methods” section. Two independent experiments were performed; error bars in graphs represent standard deviations. Values were processed by the GraphPad Prism 7 software

Finally, TB40E reactivation efficiency (Figs. 3 and 4) was compared with that achieved when THP-1 macrophages were infected directly (lytic model) (Fig. 5a, b: IE immunofluorescence pattern and titration of IE-positive cells; Fig. 5c: TCID50 viral yield titration). The results show a slightly higher infection efficiency in the latter model, coherent with the fact that input HCMV DNA used for latent infection reaches the nucleus of THP-1 monocytes with a lower efficiency than in THP-1 macrophages [38]. The TB40E reactivation efficiency was also compared with that achieved when the highly permissive MRC5 fibroblasts were infected directly (Fig. 5d, e: IE immunofluorescence pattern and titration of IE-positive cells; Fig. 5f: TCID50 viral yield titration). As expected, the highest efficiency of infection was observed in the latter model.

### Exosome analysis

The presence of exosomes was monitored in the supernatants of uninfected THP-1 monocyte-derived macrophages, TB40E-infected THP-1 monocyte-derived macrophages (reactivation model) and in the viral inoculum derived from the TB40E reactivation model applying the protocols described in the Methods section.

**Fig. 6:**
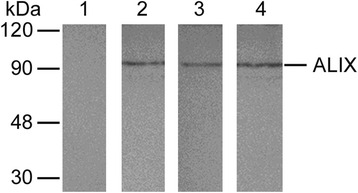
Biochemical analysis of Alix exosomal marker. After a 7 day-infection at an MOI 0.5, exosomes were purified from cell culture supernatants of infected THP-1 derived macrophages (reactivation model) and uninfected THP-1 derived-macrophages. Equal amounts (20 μg) of each sample, as well as the viral inoculum derived from the TB40E reactivation model (MOI 0.5) and a positive control (exosomes purified from COLO-1 cells), were run by SDS-PAGE and transferred to a nitrocellulose membrane, WB analysis was performed using a mouse monoclonal antibody raised against the ALIX (95 kDa) exosomal marker. The immunoreactions were revealed by AP-conjugated anti-mouse antibody. Lane 1: protein fraction obtained from the supernatant of TB40E-infected THP-1 derived-macrophages (reactivation model), as detailed in the Methods section; lane 2: protein fraction obtained from the supernatant of uninfected THP-1 derived-macrophages processed for exosome extraction, as detailed in the Methods section; lane 3: protein fraction obtained from the supernatant of infected THP-1 derived-macrophages (reactivation model) processed for exosome extraction, as detailed in the Methods section; lane 4: purified exosomes derived from the cell culture supernatant of COLO1 cells (positive control). Molecular weight markers are indicated to the left of the figure

The presence of the exosomal marker Alix was analysed by WB (Fig. 6); the results show a clear signal in the supernatants derived from uninfected (Fig. 6, lane 2) and TB40E-infected THP-1 monocyte-derived macrophages (Fig. 6, lane 3), as well as in the positive control (Fig. 6, lane 4: exosomes from COLO-1 cells). On the other hand, no signal was present in the viral inoculum derived from the TB40E reactivation model (Fig. 6, lane 1).

## Discussion

Despite being the subject of intense study over recent years, the mechanisms controlling the switch between latency and reactivation, the outcome of HCMV infection upon reactivation and the duration and severity of clinical manifestations are still unclear. Such mechanisms are likely to involve both immunological and non immunological host factors as well as viral determinants, highlighting the need for studies into the regulation of viral genome expression, genotyping and identification of potential virulence markers.

As already mentioned, these studies have been greatly hampered by the low number of peripheral blood monocytes harbouring the latent virus and by the low amount of HCMV DNA present in infected cells [28–32]. Thus, experimental models are needed able to mimick cell targets in vivo, and that can also provide more adequate quantities of cells harbouring latent or reactivated virus.

It is known that THP-1 monocytes are not permissive to HCMV lytic infection [37] and that they have been largely exploited as a HCMV latency model [36–39]. THP-1 monocyte differentiation into macrophages mediates the passage to a post-mitotic step and determines permissiveness to HCMV productive replication. In this respect, THP-1 macrophages have also been extensively used as a lytic system, by inducing differentiation into macrophages prior to infection [36, 37, 42, 58, 59].

On the other hand, a smaller number of studies have specifically addressed the use of THP-1 monocytes as a reactivation model (by inducing differentiation into macrophages after infection). However, the available data is fragmentary and does not relate to the whole lytic programme [45–48]. In most of these studies, the authors first looked at the IE gene expression. Indeed, the major IE gene proteins are known to play a pivotal role in the activation of the HCMV lytic cycle [31]. However, their expression, which is required for reactivating HCMV replication [31, 60], is not sufficient for the completion of productive infection [61–63].

Furthermore, even though few of the aforementioned authors looked at specific HCMV early and late gene expression [46–48] or provided some evidence of viral yield production [49], these approaches, never addressed at the complete development of the lytic cycle upon reactivation; this has given rise to doubts concerning the use of THP-1 monocytes as a true latency-reactivation model. Indeed, more recently Shen and collaborators [40] have highlighted that this cell line should not be considered as a true latency model because reactivation to produce infectious progeny had not been clearly demonstrated until now. In this regard, Yee and collaborators [49] observed that upon phorbol ester/hydrocortisone-induced differentiation of latently infected THP-1 monocytes, an infectious progeny could be demonstrated by co-cultivation with human foreskin fibroblasts. To this respect, it should be noted that the Authors used the Towne strain (i.e. a serially passaged, fibroblast adapted virus) for THP-1 cells infection at very high MOI and that these experimental conditions are quite far from those occurring in vivo (circulating strains able to infect monocytes and macrophages; low infection efficiency in monocytes). Moreover, considering the possible role of exosomes as a vehicle of intercellular exchange of viral components, it is worth noting that the co-cultivation of THP-1 cells with human fibroblasts does not constitute unequivocal evidence of infectious virus production from THP-1 cells. The transfer of viral proteins or transcripts in this way could indeed enhance viral infection in recipient cells, even via a viral receptor-independent, envelope-independent mode of infection [64, 65]; this rightly raises some doubts regarding the ability of HCMV to complete its lytic cycle in THP-1 cells.

In order to address and clarify this issue, the present study investigated for the first time the use of THP-1 cells as a model of true viral reactivation by analysing both the start and the completion of the lytic cycle up to the infectious progeny production, and compared the events with those occurring in the same cells employed as a latency model.

We first wanted to verify whether the input efficiency of the infecting HCMV (using the endotheliotropic TB40E strain) and its nuclear translocation were similar in the latency and reactivation models, and whether they were influenced by the trigger of viral replication. This was done by checking the amount of viral DNA at 30 h p.i., i.e. a time period that was sufficiently short to exclude a significant amplification of the HCMV genome [37]. This allowed us to demonstrate the comparable efficiency of viral DNA translocation into cell nuclei in both infection models at 30 h p.i. and to confirm that increased HCMV DNA amounts only observed in the reactivation model (mostly at 7 days p.i.) were actually due to the viral genome replication as a consequence of activation of the lytic programme. These data were also confirmed by repeating the experiments using a UV-inactivated virus. On the other hand, a decrease in viral DNA amounts was observed in the latent model over time. This could be explained by the partial loss of latent HCMV genomes–present as episomal DNA in cell nuclei–in proliferating cells, as previously seen to occur in THP-1 monocytes [48]. According to the DNA data, viral transcripts representative of the different phases of the lytic cycle were only found after 7 days of infection in the reactivation model. A very faint signal for the IE1 transcript was observed in the latency model at an MOI of 0.5, possibly due to a very low amount of spontaneously differentiated THP-1 monocytes which should allow the lytic cycle to start [37].

Considering that the presence of transcripts from candidate latency-associated genes should be demonstrated in cells latently infected with HCMV in the absence of productive infection transcripts [66], we also verified the presence of the anti-sense cytomegalovirus latency-associated transcript, encoded from anti-sense strands of the major immediate-early (MIE) region of the HCMV genome in the latency model [47]. A fainter signal was also observed in the reactivation model, consistent with the notion that cytomegalovirus latency-associated transcripts could also be present during lytic infection [47].

Finally, we looked at the HCMV progeny derived from the reactivation model by analysing the presence of infectious virus in the cell culture medium of THP-1 monocytes differentiated into macrophages after TB40E infection at 7 days p.i.. The results of the immunofluorescence patterns of immediate-early and late viral antigens and TCID_50_ titres performed on MRC5 fibroblasts clearly confirmed the presence of a THP-1-derived viral yield. As expected, the reactivation efficiency of the endotheliotropic strain TB40E was far higher than that observed with the highly passaged laboratory Towne strain, but lower than that obtained with THP-1 monocyte-derived macrophages (used as a model of lytic infection) or the highly permissive MRC5 fibroblasts.

We also monitored the presence of exosomes in the viral inoculum derived from the TB40E reactivation model. The results from this set of experiments clearly show the absence of any signal in the inoculum derived from the TB40E reactivation model, thus excluding the possible delivery of viral components via exosomes to the MRC5 permissive model.

## Conclusions

Our data demonstrate, for the first time, the effectiveness of THP-1 cells as a good reactivation model, showing clear evidence of the development and completion of the lytic cycle, leading to the production of viral progeny. Moreover, the results obtained in the present study, using progressively increasing MOI (although always low) confirm the possibility of using this “switch” model with higher concentrations of the input virus in order to obtain a larger number of cells harbouring latent or reactivated HCMV, thus making studies into the regulation of viral genome expression easier to implement.

Understanding the mechanisms governing latency establishment and reactivation could also pave the way to new therapeutic approaches, making the development of in vitro experimental models for latency/reactivation studies of utmost importance.

## Additional files

Additional file 1: Flow cytometry characterization of CD11b and CD14 differentiation markers in uninfected THP-1 monocytes and THP-1 monocyte-derived macrophages. A–For the quantitative evaluation of cells expressing CD11b and CD14 differentiation markers in THP-1 monocytes and THP-1 macrophages at 4 and 6 days after TPA-differentiation the immunofluorescence reaction was performed as described in the Methods section. B–Analysis of the Mean Fluorescence Intensity (MFI) values of CD11b and CD14 in uninfected THP-1 monocytes and THP-1 macrophages at 4 and 6 days after TPA differentiation. MFI values were subtracted by the MFI of isotype control antibody-stained cells. The results were expressed as percentage of CD11b and CD14 positive THP-1 monocytes. Analyses were done by gating viable cells. Two independent experiments were performed; error bars in graphs represent standard deviations. Values were processed by the GraphPad Prism 7 software. (PDF 613 kb)
Additional file 2: THP-1 macrophage (lytic model) infection with TB40E progeny derived from the THP-1 reactivation model. THP-1 macrophages (lytic model) were infected with the cell culture supernatant derived from the THP-1 reactivation model (as detailed in the Methods section), which had been infected with the TB40E strain at MOI of 0.5 (panels a, a’), 0.25 (panels b, b’) or 0.125 (panels c, c’) for 7 days; panels d, d’: uninfected cells. A–At 24 h p.i., THP-1 macrophages were fixed and labelled with an anti-IE antibody (“IE-positive THP-1 macrophages from the THP-1 reactivation model”). B–at 72 h p.i. they were fixed and labelled with an anti-pp65 antibody (“pp65-positive THP-1 macrophages from THP-1 reactivation model”). Bar: 25 μm. (PDF 1315 kb)

## Abbreviations

AP: Alkaline phosphatase
CLT: Cytomegalovirus latency-associated transcripts
Cyt: Cytoplasm
DAPI: 4′,6-diamidino-2-phenylindole
E-MEM: Earle’s modified Minimum Essential Medium
FBS: Foetal bovine serum
FITC: Fluorescein isothiocyanate
GAPDH: Glyceraldehyde-3-phosphate-dehydrogenase
HCMV: Human cytomegalovirus
Lys: Lysate
MOI: Multiplicity of infection
MW: Molecular weight
Nuc: Nuclei
p.i.: post infection
PBS: Phosphate buffer saline
PFU: Plaque forming units
RPMI: Roswell Park Memorial Institute
RT: Reverse transcription
SDS-PAGE: Sodium dodecyl sulfate-polyacrylamide gel electrophoresis
TCA: Trichloroacetic acid
TPA: 12-*O*-tetradecanoylphorbol-13-Acetate
WB: Western blotting
