# Supplementary figures and images for: Human cytomegalovirus reactivation from latency: validation of a “switch” model in vitro
Source: Virol J. 2016 Oct 22;13:179. doi: 10.1186/s12985-016-0634-z (PMC5075216; doi:10.1186/s12985-016-0634-z)

**a**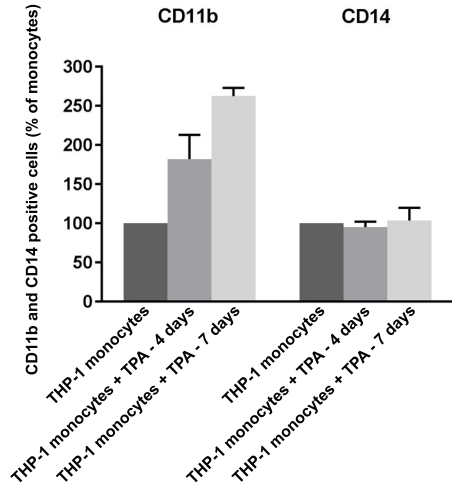**b**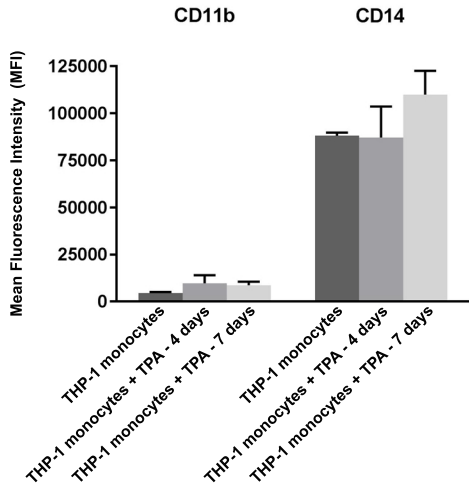

Supplement: Additional file 1: — Flow cytometry characterization of CD11b and CD14 differentiation markers in uninfected THP-1 monocytes and THP-1 monocyte-derived macrophages. A–For the quantitative evaluation of cells expressing CD11b and CD14 differentiation markers in THP-1 monocytes and THP-1 macrophages at 4 and 6 days after TPA-differentiation the immunofluorescence reaction was performed as described in the Methods section. B–Analysis of the Mean Fluorescence Intensity (MFI) values of CD11b and CD14 in uninfected THP-1 monocytes and THP-1 macrophages at 4 and 6 days after TPA differentiation. MFI values were subtracted by the MFI of isotype control antibody-stained cells. The results were expressed as percentage of CD11b and CD14 positive THP-1 monocytes. Analyses were done by gating viable cells. Two independent experiments were performed; error bars in graphs represent standard deviations. Values were processed by the GraphPad Prism 7 software. (PDF 613 kb) [file 12985_2016_634_MOESM1_ESM.pdf]
