# Supplementary material for: Human cytomegalovirus reactivation from latency: validation of a “switch” model in vitro
Source: Virol J. 2016 Oct 22;13:179. doi: 10.1186/s12985-016-0634-z (PMC5075216; doi:10.1186/s12985-016-0634-z)
Supplement: Additional file 2: — THP-1 macrophage (lytic model) infection with TB40E progeny derived from the THP-1 reactivation model. THP-1 macrophages (lytic model) were infected with the cell culture supernatant derived from the THP-1 reactivation model (as detailed in the Methods section), which had been infected with the TB40E strain at MOI of 0.5 (panels a, a’), 0.25 (panels b, b’) or 0.125 (panels c, c’) for 7 days; panels d, d’: uninfected cells. A–At 24 h p.i., THP-1 macrophages were fixed and labelled with an anti-IE antibody (“IE-positive THP-1 macrophages from the THP-1 reactivation model”). B–at 72 h p.i. they were fixed and labelled with an anti-pp65 antibody (“pp65-positive THP-1 macrophages from THP-1 reactivation model”). Bar: 25 μm. (PDF 1315 kb) [file 12985_2016_634_MOESM2_ESM.pdf]

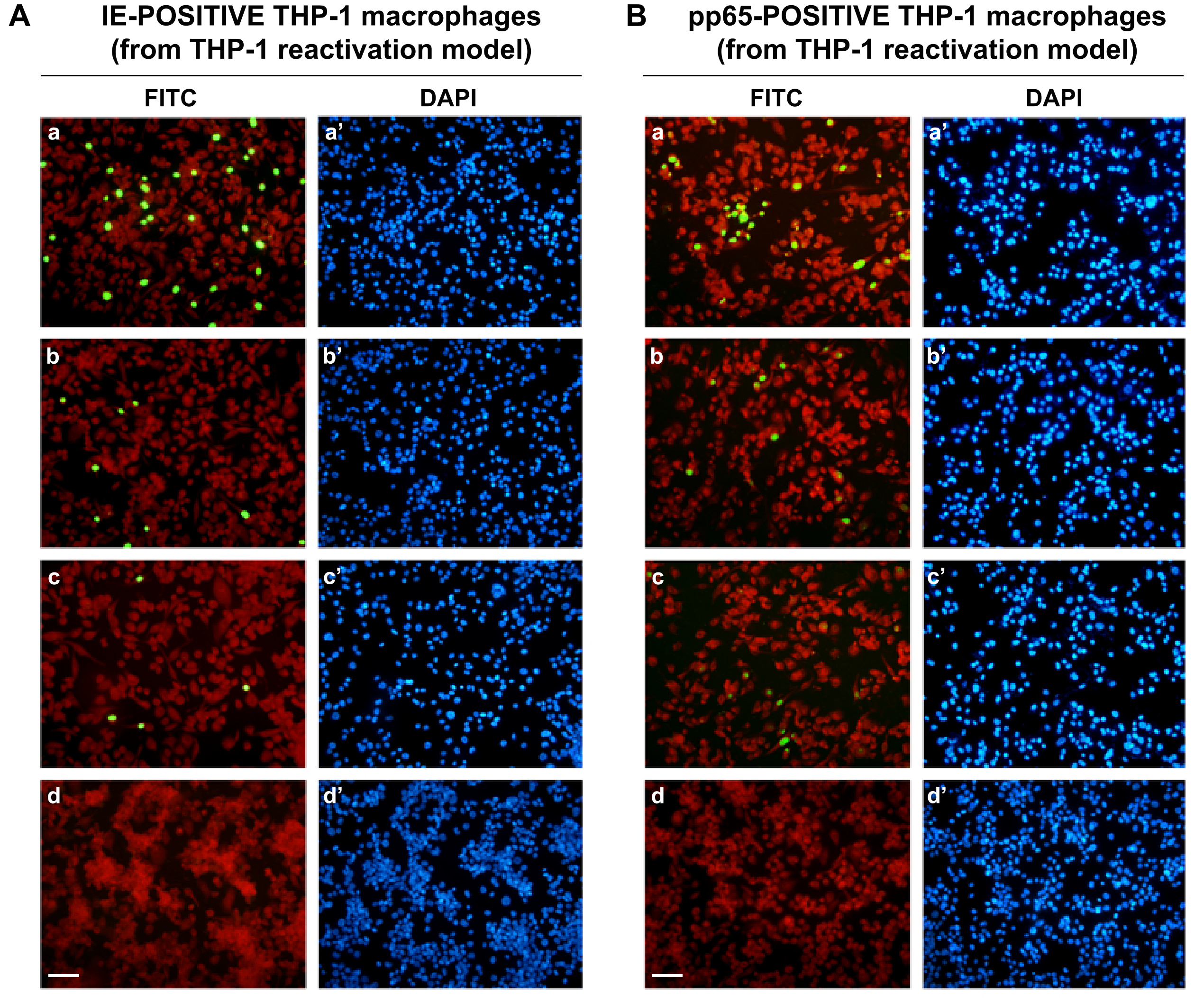
A
IE-POSITIVE THP-1 macrophages
(from THP-1 reactivation model)
FITC
DAPI
a
a'
b
b'
c
c'
d
d'
B
pp65-POSITIVE THP-1 macrophages
(from THP-1 reactivation model)
FITC
DAPI
a
a'
b
b'
c
c'
d
d'
